# Contextualization of drug-mediator relations using evidence networks

**DOI:** 10.1186/s12859-017-1642-8

**Published:** 2017-05-31

**Authors:** Hai Joey Tran, Gil Speyer, Jeff Kiefer, Seungchan Kim

**Affiliations:** 1 Integrated Cancer Genomics Division, The Translational Genomics Research Institute, Phoenix, AZ 85004 USA; 2 Department of Electrical and Computer Engineering, Roy G. Perry College of Engineering, Prairie View A&M University, Prairie View, TX 77446 USA

**Keywords:** Precision medicine, EDDY, CTRP, Gene regulatory networks, Drug development, Biochemical pathways

## Abstract

**Background:**

Genomic analysis of drug response can provide unique insights into therapies that can be used to match the “right drug to the right patient.” However, the process of discovering such therapeutic insights using genomic data is not straightforward and represents an area of active investigation. EDDY (Evaluation of Differential DependencY), a statistical test to detect differential statistical dependencies, is one method that leverages genomic data to identify differential genetic dependencies. EDDY has been used in conjunction with the Cancer Therapeutics Response Portal (CTRP), a dataset with drug-response measurements for more than 400 small molecules, and RNAseq data of cell lines in the Cancer Cell Line Encyclopedia (CCLE) to find potential drug-mediator pairs. Mediators were identified as genes that showed significant change in genetic statistical dependencies within annotated pathways between drug sensitive and drug non-sensitive cell lines, and the results are presented as a public web-portal (EDDY-CTRP). However, the interpretability of drug-mediator pairs currently hinders further exploration of these potentially valuable results.

**Methods:**

In this study, we address this challenge by constructing evidence networks built with protein and drug interactions from the STITCH and STRING interaction databases. STITCH and STRING are sister databases that catalog known and predicted drug-protein interactions and protein-protein interactions, respectively. Using these two databases, we have developed a method to construct evidence networks to “explain” the relation between a drug and a mediator.

**Results:**

We applied this approach to drug-mediator relations discovered in EDDY-CTRP analysis and identified evidence networks for ~70% of drug-mediator pairs where most mediators were not known direct targets for the drug. Constructed evidence networks enable researchers to contextualize the drug-mediator pair with current research and knowledge. Using evidence networks, we were able to improve the interpretability of the EDDY-CTRP results by linking the drugs and mediators with genes associated with both the drug and the mediator.

**Conclusion:**

We anticipate that these evidence networks will help inform EDDY-CTRP results and enhance the generation of important insights to drug sensitivity that will lead to improved precision medicine applications.

## Background

Response to a drug within a cancer cell involves complex protein signaling processes dependent on the molecular context of the cell and the properties of the individual drug. Transcriptomic data of cancer cell lines coupled with drug response data constitute a rich data set to study drug response and underlying molecular mechanisms. However, the scale of these data presents many unique analytical challenges. Data driven approaches generate a large number of associations and observations that can stand as testable hypotheses. We have utilized this genomic data in the development of a unique algorithm, EDDY (Evaluation of Differential DependencY) [1], that uses gene expression data and conditions to construct differential dependency networks of given gene sets [2] between the conditions.

Through statistical interrogation of gene dependencies within an annotated pathway from a gene network catalog such as REACTOME [3], EDDY repeatedly constructs networks from resampled RNAseq data for each of two conditions. The divergence between the two resulting distributions of networks can then be assessed for significance through permutation test.

EDDY was used with data integrated from the Cancer Therapeutics Response Portal (CTRP) and the Cancer Cell Line Encyclopedia (CCLE) [4–6]. The CTRP dataset contains drug-response measurements for more than 400 small-molecules applied to CCLE cell lines. For each compound, cell lines were classified as either sensitive or non-sensitive for analysis by EDDY in order to identify: 1) pathways enriched with differential dependency between sensitive and non-sensitive cell lines for each compound, and 2) differential dependency networks (DDNs) that capture how gene dependency was rewired. We then identified the genes, termed “mediators”, that played a significantly different role (based on gene dependency networks) between cell lines that were sensitive to a drug and cell lines that were non-sensitive. The details of this analysis and the results have been published in a separate article [7]. We will refer to this analysis as EDDY-CTRP throughout this manuscript.

We predict that these drug-mediator pairs have potential as testable hypotheses on drug sensitivity and discovery of novel drug targets. However, the interpretability of these results serves as a bottleneck on further experimental validation. Currently, to further understand these drug-mediator pairs, a researcher must manually search through current literature, which is often a slow and inefficient process. Furthermore, the sheer volume of peer-reviewed research prevents researchers from reliably finding the most pertinent data to inform these hypotheses.

There are currently multiple databases that can help alleviate this problem by cataloging drug-protein interactions and protein-protein interactions, such as Pathway Commons, STITCH, STRING and BioGrid [8–11]. Currently though, these databases have no easy way of exploring possible drug-mediator relationships. Some of these databases allow for researchers to query for both a drug and a gene but the presentation of the relationships make no effort to show how the drug and gene may be related and often end up displaying many irrelevant genes.

In this study, we attempt to improve the interpretability of the EDDY-CTRP results by contextualizing the drug-mediator pairs with current research using evidence networks generated from the STRING and STITCH knowledge-bases. For this study, we define evidence networks as sub-networks of knowledge-bases that present the most relevant intermediate nodes that have established functional associations with the drug and mediator based on prior research. We chose to use STRING and STITCH as our knowledge-bases for their comprehensive volume of data and for their distinction of different types of evidence into separate association scores.

## Methods

### STRING and STITCH databases

The edges of the STRING and STITCH network were downloaded as flat files and were reconstructed into networks. Each edge in the STRING and STITCH database included scores based on how much evidence established them and how compelling they are. These scores were further broken down into sub-scores based on what type of source the evidence came from. Specific descriptions of evidences for the edges were downloaded separately as a PostGreSQL database, which was later queried against to annotate the generated evidence networks.

In order to maximize the accuracy of chemical matching, all drugs were reduced to SMILES strings (a string representation of a compound’s molecular structure). From their SMILES strings, drugs were then hashed into their respective InChIKeys. InChIKeys are encoded strings that are unique to a chemical structure. The InChIKeys were then queried against the STITCH database, which also stores InChIKeys for most of its catalogued chemicals. The use of InChIKeys helped minimize the number of drugs that are matched with other drugs that may use the same aliases. Like STITCH, all InChIKeys were reduced to their non-stereospecific forms, and all salt forms of compounds were considered as one compound. All InchiKey conversions were done using the RDKit open-source cheminformatics software package [12].

### Construction of evidence networks

The evidence network is a network made of a compound as a starting node, a mediator as a terminal node, and a set of genes connecting the two. It suggests an evidence-supported explanation for why and how the mediator gene is interacting with the compound.

Evidence networks were generated using a modified Yen’s *K* Shortest Paths [13] algorithm with a weight function of *w*_*edge*_ = 1 − *S*_*edge*_, where *S*_*edge*_ is an evidence score described above. Hence, edges with higher scores would be preferred over edges with lower scores (all scores range from 0 to 1). To generate the evidence networks, shortest paths between a compound and a mediator were iteratively found and added to the network until there were no more paths from the compound to the mediator or until there were at least *N* distinct nodes in the sub-network, where *N* is some arbitrary threshold. *N* was not a strict floor, as sometimes the last path added to the sub-network would add two or more distinct nodes pushing the total number of distinct nodes over the threshold. Instead, *N* was used simply as a stopping condition and was chosen in order to prevent generation of evidence networks that would be too overwhelming for users to interpret. Dijkstra’s Shortest Path algorithm [14] with a Fibonacci heap [15] was used as the supporting shortest path algorithm in the modified Yen’s *K*-Shortest Paths algorithm, as shown in Algorithm 1.

The motivation for using the *k*-shortest paths between the drug and gene is that researchers will most likely look for relationships between the drug and gene that have the best evidence, i.e., the highest overall evidence score, to support them. However, to account for high redundancy in signaling pathways, the evidence network is constructed to allow the researcher to see more than just the most direct path from the drug to the gene. Multiple shortest paths are found and merged together in order to form evidence networks that allow the researcher to explore multiple different possible relationships.
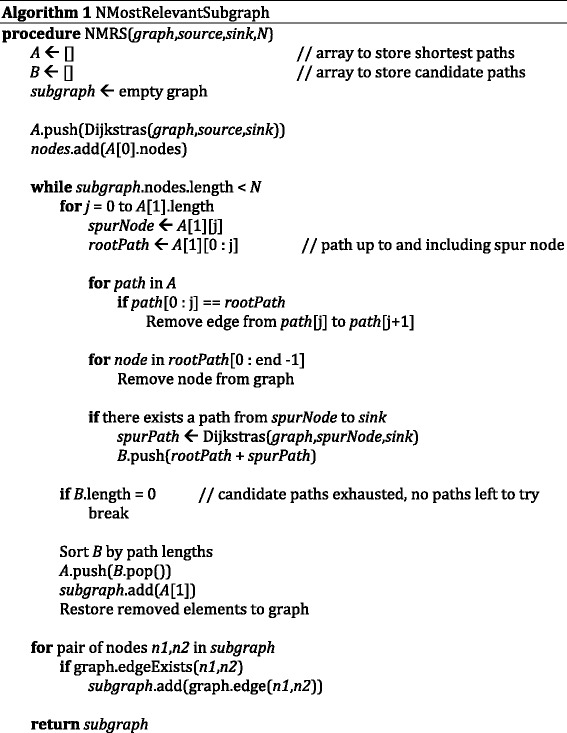


### Extensions of evidence networks

In addition to the methods described above, two additional extensions of evidence networks were explored. One extension included exploring the use of single-source evidence networks constructed by including a mediator and *k* number of closest drugs found in the STITCH/STRING database. These types of evidence networks were initially employed in the situation where EDDY-CTRP identified a drug-mediator pair but the drug could not be found in the STITCH database. If a homologous compound could be identified, evidence nets could then be constructed using the homolog as a substitute for the original drug. We can envision a variety of additional applications for this extension. For example, in the situation where a mediator is identified and it appears to play a role in resistance to a compound, the identification of drugs that are known to interact, directly or indirectly, with this mediator might then suggest a possible combination therapy with the original compound.

In order to preserve the pathway-specific context of the mediator, a second extension of evidence networks was explored which constructed evidence subnetworks for each direct neighbor of a mediator in the original differential dependency network. The direct neighbors of a mediator were defined as genes that were directly connected to the mediator in either of the condition-specific dependency networks for a given mediator. To merge the direct neighbor evidence subnetworks, a set was created containing all distinct nodes from each subnetwork with the addition of the original mediator. Then, for each pair of nodes in this merged set, we checked the STITCH/STRING network to see if an edge existed between the nodes and included it if it did. If there were no paths between a direct neighbor and the drug, the direct neighbor was still included as a node in the network. Since this extension required building an evidence subnetwork for each direct neighbor, the resulting network, while potentially increased in density, often related more clearly to the original DDN and, thereby, its associated pathway. This “pathway-weighted” contextualization aims to extend the filtering of evidence networks, relating the biological context of the mediator’s original DDN to the compound and its known targets.

## Results

### EDDY-CTRP evidence networks

EDDY-CTRP analysis identified 26,822 drug-mediator pairs. Among those pairs, 19,222 (75%) of them consisted of a drug and gene that were contained within the STRING and STITCH databases. Evidence network analysis found 14,415 (70%) pairs with evidence network with 3 or less number of intermediate genes. Evidence networks were constructed for 14,415 (70%) of the drug-mediator pairs. Evidence networks were limited to having at most three intermediate genes between the compound and mediator since compound and mediators connected with more than three intermediate genes likely had little relevance to one another. These evidence networks were integrated into the main EDDY-CTRP portal as a searchable table. The distribution of number of intermediate genes for each drug-mediator pair is shown in Table 1.

**Table 1:** Distribution of the number of intermediate genes in shortest path between compound and mediator pair

	Direct targets	Indirect targets		
		# of intermediate genes in shortest path		
		1	2	3
# of pairs	102	988	3,410	9,915

We note that 102 evidence networks indeed were direct compound and mediator relations, among which only 34 of them were intended targets defined in the CTRP data and annotation. This indicates STITCH contains drug-target relations that were not included in the CTRP database, but EDDY-CTRP analysis was able to discover those relations. Most of these evidence networks were for drug-mediator pairs where mediators were not direct targets of the drug (according to the CTRP annotation) but had some known functional association to the drug (based on STITCH/STRING database).

**Fig. 1:**
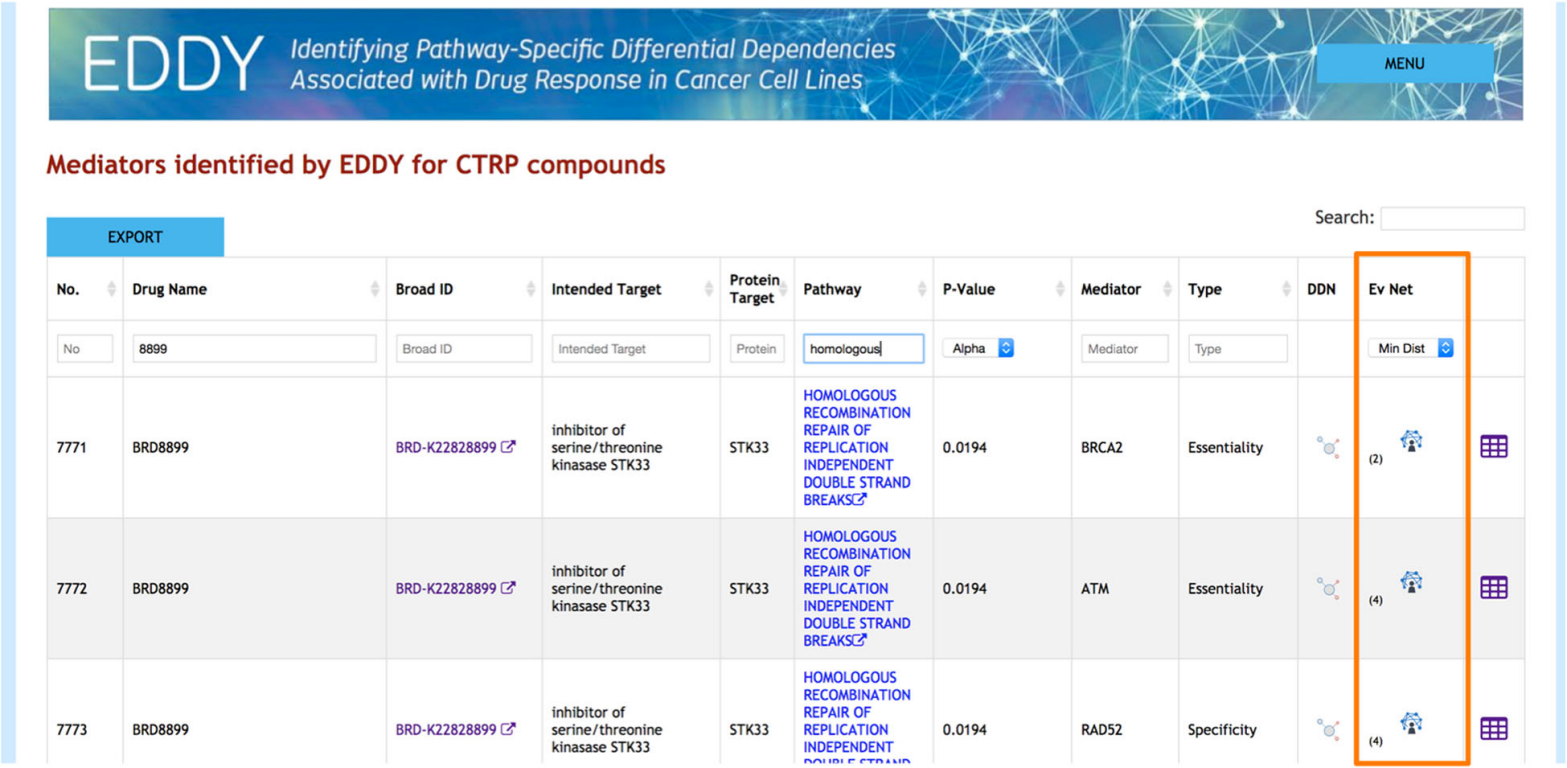
Screenshot from EDDY-CTRP portal (http://biocomputing.tgen.org/software/EDDY/CTRP) showing access point to evidence networks in mediator table “Ev Net” column (highlighted). The user can filter by compound – mediator distance within the network

**Fig. 2:**
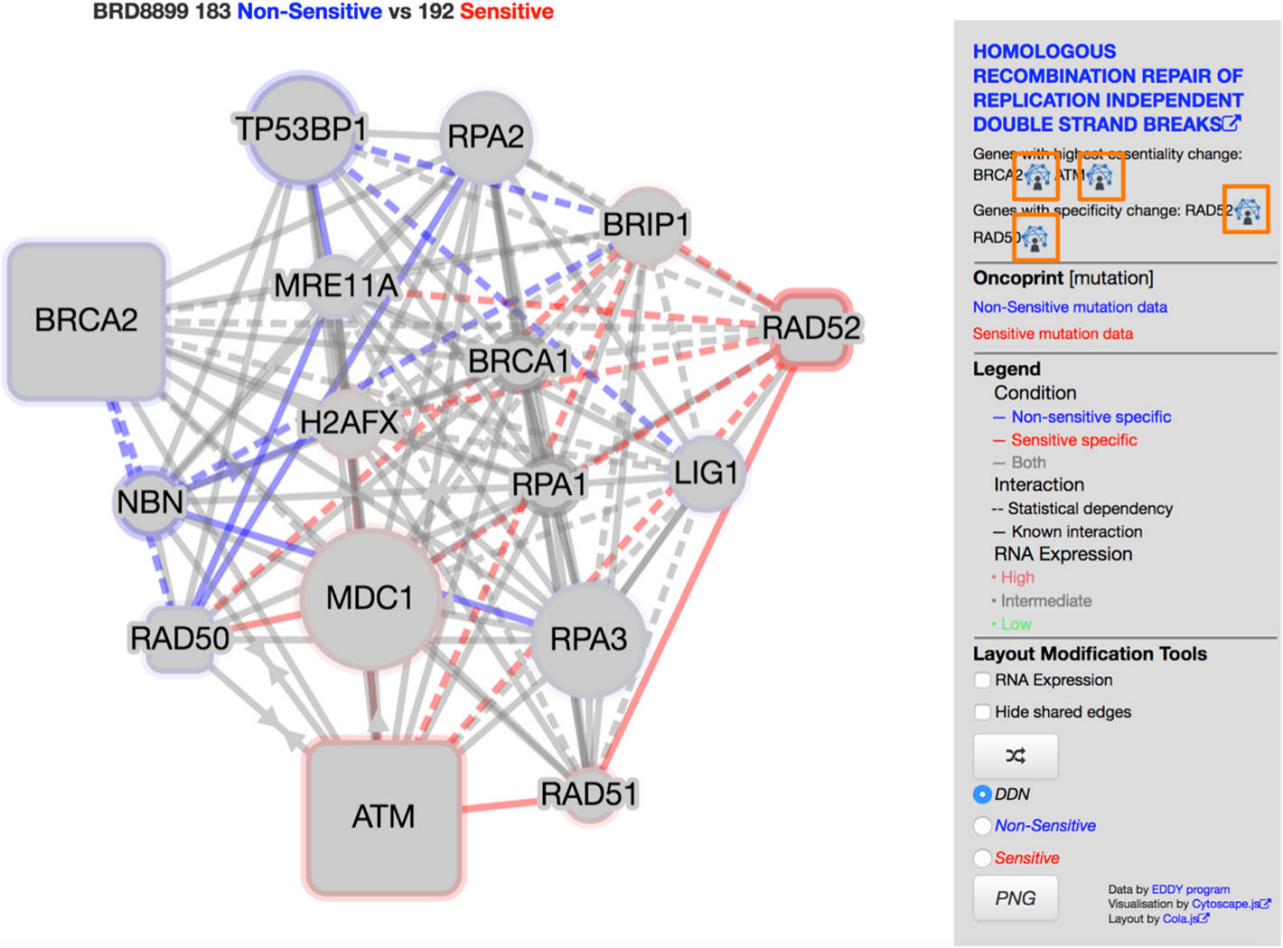
Screenshot from EDDY-CTRP portal (http://biocomputing.tgen.org/software/EDDY/CTRP) showing access point to evidence networks in DDN. Symbols following each mediator listed in upper right panel (highlighted in *orange*) link directly to evidence networks

**Fig. 3:**
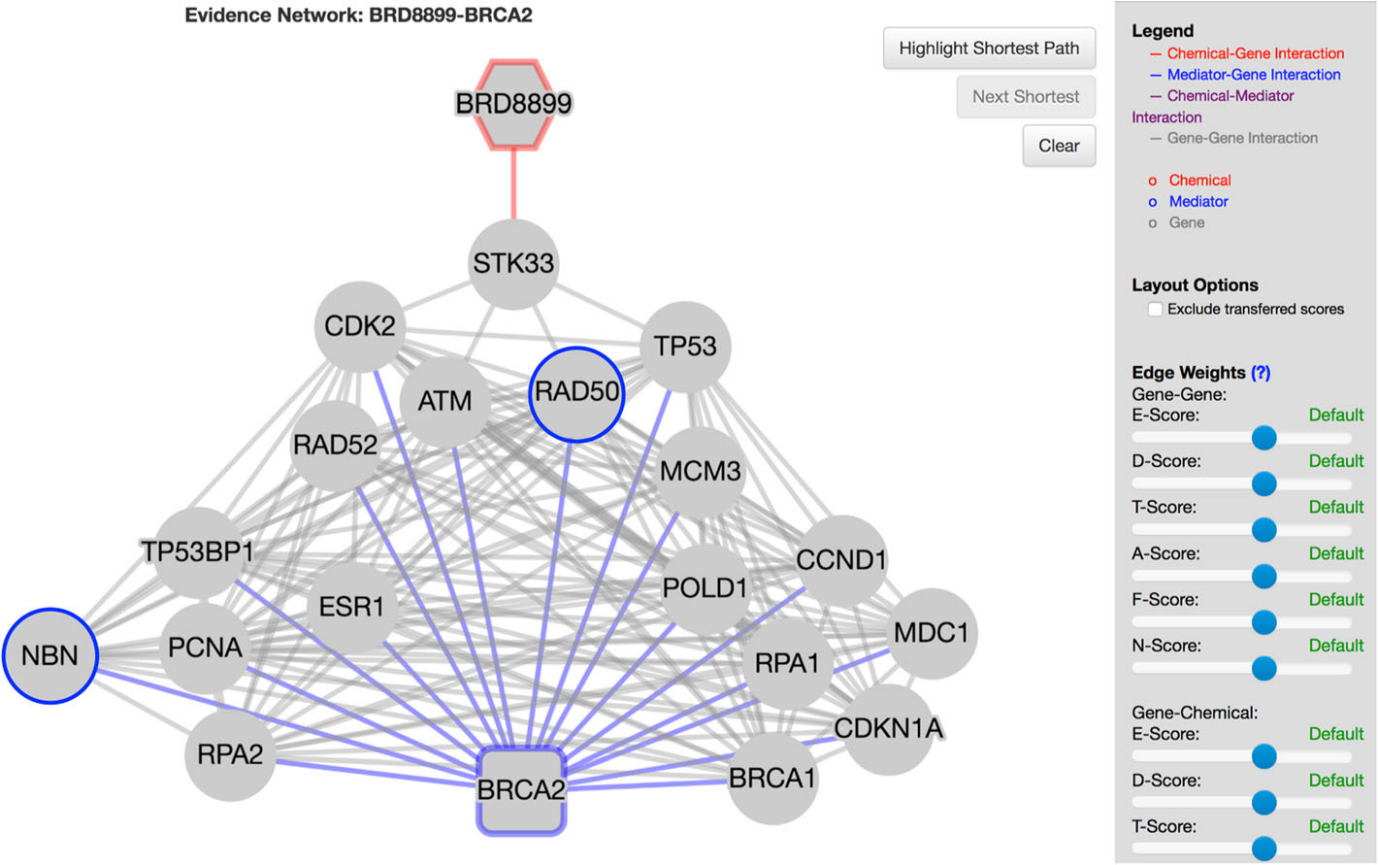
Screenshot from EDDY-CTRP portal (http://biocomputing.tgen.org/software/EDDY/CTRP) showing “pathway-weighted” evidence network for BRD-8899 – BRCA2 compound mediator pair. Nodes connected to BRCA2 by non-sensitive condition-specific edges in DDN are highlighted in *blue*

To allow an interactive presentation of the evidence networks, a web-portal was created using the JavaScript library Cytoscape.js [16] and built into the main EDDY-CTRP web-portal (http://biocomputing.tgen.org/software/EDDY/CTRP). These evidence networks can be accessed at two places in the portal. First, in the compound mediator table, (Fig. 1), the evidence nets, where available, are linked in the penultimate “Ev Net” column. Second, in the DDN view (Fig. 2), mediators are listed in the upper right panel. A symbol follows each gene name where an evidence network is available. Once clicked, the evidence net view (Fig. 3) features: 1) the drug and genes of interest highlighted along with their functional associations with other genes, 2) the opacity of each edge mapped to its overall score, which is determined by the evidence that characterizes it, 3) brief information for each edge describing the evidence establishing it, and 4) if further information is desired, links to the STITCH or STRING database for each edge, which gives further details.

To help the user find the most direct paths from the drug to the gene of interest, the most direct path can be highlighted by clicking the on-screen button. To explore alternative paths, the user can also highlight the next shortest paths by repeatedly clicking the on-screen button labeled “Next Shortest.” Data channel weights are also included in the interface to allow users to weight different types of evidence based on their preferences. For example, a user who does not find text-mining evidence to be compelling can prioritize text mining scores to “LOW” or “NONE,” and the edge weights and shortest paths will be recalculated and redrawn accordingly.

### Evidence network corroborates DAPK3’s role as a mediator for TG-101348

**Fig. 4:**
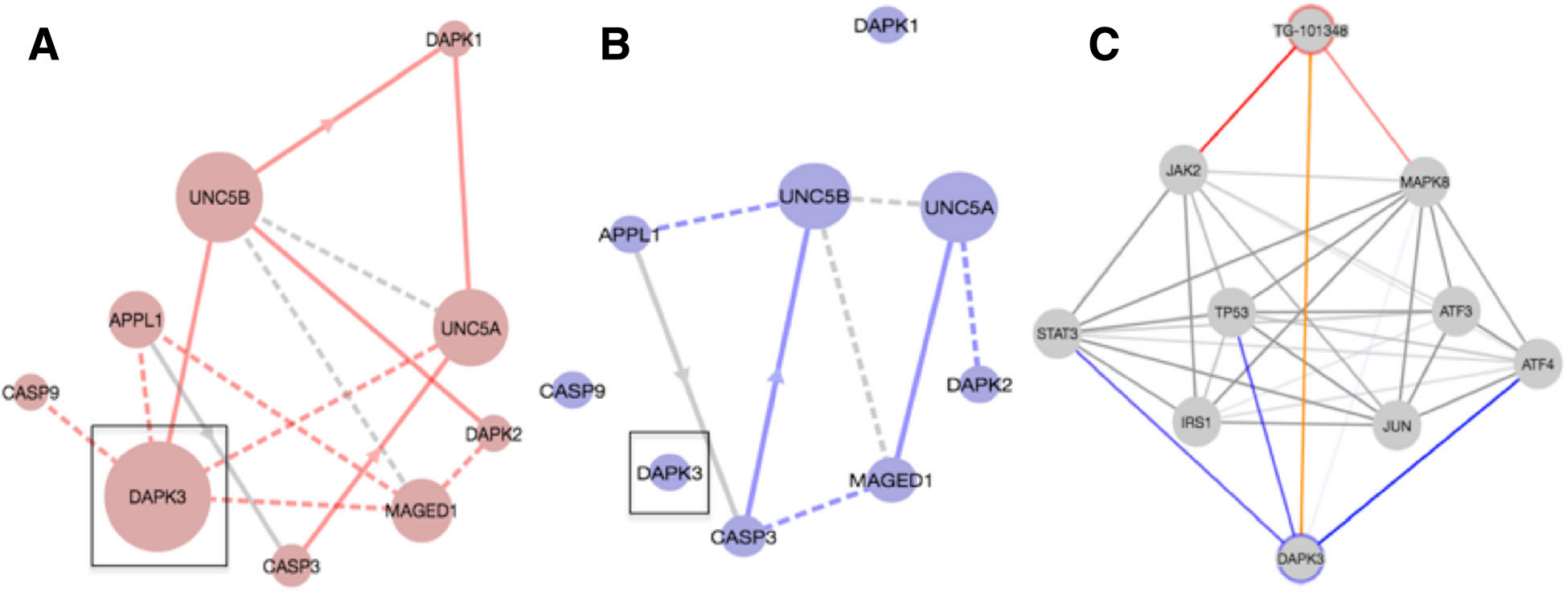
**a** Condition-specific dependency network (CDN) for TG-101348 for drug-sensitive cell lines. Dashed lines represent statistical dependencies while solid lines represent known interactions. Size of nodes represents node essentiality. **b** CDN for TG-101348 for drug-insensitive cell lines. **c** Evidence network for the TG-101348 – DAPK3 drug-mediator pair. All edges represent a known association based from the STRING/STITCH databases. *Blue* edges represent mediator-gene associations. *Red* edges represent drug-gene associations. The *yellow* edge represents a direct drug-mediator association

TG-101348 was developed as a selective inhibitor of JAK2 kinase for the treatment of myeloproliferative disorder. EDDY identified the "ROLE OF DCC IN REGULATING APOPTOSIS" pathway as an altered differential dependency network for TG-101348. The gene product of DAPK3 was a mediator in this pathway due to high change of essentiality between the condition-specific dependency networks. In TG-101348-sensitive cell lines, DAPK3 is highly connected in the network (Fig. 4a), which likely indicates that DAPK3 plays a central role in a functioning apoptotic network. In the resistant cell lines, however, DAPK3 is not connected to the rest of the network (Fig. 4b), indicating that DAPK3 likely plays a role in TG-101348 sensitivity. The evidence network built for TG-101348 - DAPK3 supports this hypothesis by showing a direct association between TG-101348 and DAPK3 from the STITCH database (Fig. 4c). Indeed the evidence link was from a study that showed TG-101348 can inhibit the kinase activity of DAPK3, indicating that TG-101348 actually does target DAPK3 in addition to JAK2. Additionally, an association between the downstream JAK2 modulator and DAPK3 was revealed suggesting further signaling interactions targeted by TG-101348 [17]. This example reveals how the evidence network provides further contextual information regarding the possible mechanisms of how mediators selected in the EDDY analysis function to alter individual drug responses.

### BRD-8899:BRCA2 evidence network discovers novel interaction between STK33 and DNA repair mechanism

**Fig. 5:**
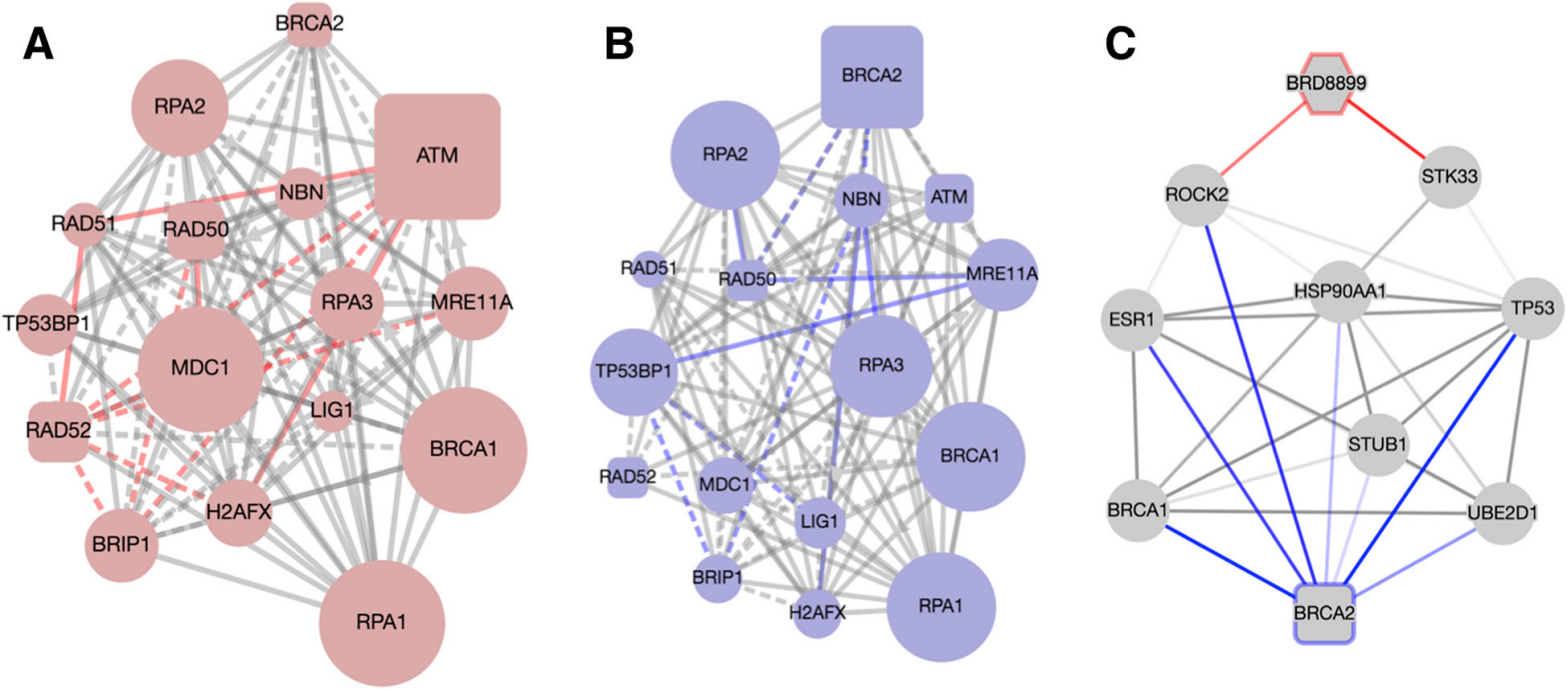
**a** Condition-specific dependency network (CDN) for BRD-8899 for drug-sensitive cell lines. Dashed lines represent statistical dependencies while solid lines represent known interactions. Size of nodes represents node essentiality. **b** CDN for BRD-8899 for drug-insensitive cell lines. **c** Evidence network for the BRD-8899 – BRCA2 drug-mediator pair. All edges represent a known association based from the STRING/STITCH databases. *Blue* edges represent mediator-gene associations. *Red* edges represent drug-gene associations

BRD-8899 is employed as a small-molecule inhibitor of serine/threonine kinase (STK33) activity. The “HOMOLOGOUS RECOMBINATION REPAIR OF REPLICATION INDEPENDENT DOUBLE STRAND BREAKS” pathway was identified by EDDY as significantly rewired. The rewiring of this pathway could imply a heretofore unknown mechanism related to STK33 and DNA repair. Additionally, if STK33 plays no role in homologous recombination (HR) repair, this could suggest a possible secondary target for BRD-8899. Further, in examining the rewiring between the condition-specific pathways, the mediator gene BRCA2 in this pathway can be seen to change in essentiality, but this time having a more important role in the non-sensitive cell lines (Fig. 5a and b). This suggests that the loss of BRCA2 signaling in the double strand break repair mechanism may play a role in BRD-8899 sensitivity. We can develop our understanding through examination of the evidence net (Fig. 5c), where we can see that STK33 does indeed interact with TP53 and heat shock protein (HSP) 90AA1 which both interact with BRCA2. Thus, through identifying a role for BRCA2 in BRD-8899 resistance, we could propose an experiment employing a combination therapy on non-sensitive cell lines targeting BRCA2, such as a PARP inhibitor.

### Single-source evidence network of CCNH discovers potential alternative compounds for OSI-027

**Fig. 6:**
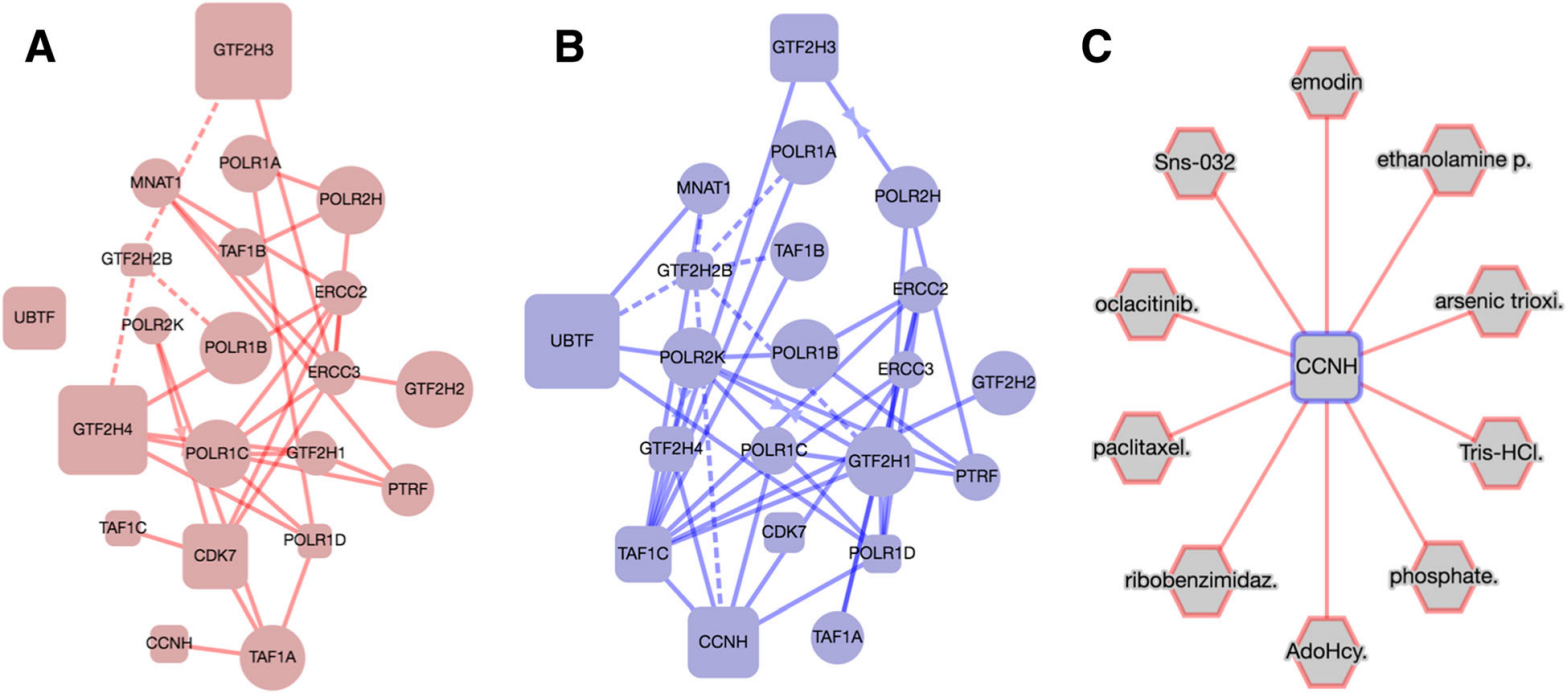
**a** Condition-specific dependency network (CDN) for OSI-027 for drug-sensitive cell lines. Dashed lines represent statistical dependencies while solid lines represent known interactions. Size of nodes represents node essentiality. **b** CDN for OSI-027 for drug-insensitive cell lines. Shared (*grey*) edges are hidden for clarity in both CDNs. **c** Single-source evidence network for the CCNH mediator. All edges represent a known association based from the STRING/STITCH databases

EDDY analysis of cell lines sensitive and non-sensitive to OSI-027, an MTOR inhibitor, discovered several significant pathways, including “RNA POL I TRANSCRIPTION TERMINATION,” as well as mediators associated with this network. As no mediator pairings with OSI-027 produced evidence networks through STITCH/STRING, the mediators were then analyzed using the single-source contextualization extension described above. In the condition-specific networks shown in Fig. 6, the essentiality of one of the mediators, Cyclin H (CCNH, at bottom of figure) changes dramatically between sensitive and non-sensitive cell lines, suggesting that CCNH plays a more important role in the non-sensitive network. A possible hypothesis for experiment could involve the perturbation of CCNH in non-sensitive cell lines and then assessing the efficacy of OSI-027. The single-source queries for CCNH discover several compounds of potential interest. One of these, paclitaxel, has been shown in cancer cell line experiments to improve in efficacy when used in combination with an MTOR-inhibitor [18]. Two other connections, SNS-032, a cycline-dependent kinase inhibitor, and emodin, have been shown to inhibit MTOR activity [19, 20].

### Pathway-weighted contextualization of the BRD-8899:BRCA2 develops additional features of possible STK33 role in homologous repair

Returning to the BRD-8899 – BRCA2 mediator pair, nearest neighbors from the original DDN have been supplemented leading to greater contextual clarity (Fig. 3), compared to Fig. 5c. The ROCK2 association from the original evidence network, which did not relate to the homologous repair pathway, is no longer present, but new connections related to RAD52, RAD50, NBN and ATM have been added. ATM and RAD50 have direct links to STK33, the target of the compound, and RAD52 and NBN link to STK33 via CDK2. These additional links suggest possible means by which STK33, and thereby BRD-8899, influences homologous repair. As an essentiality mediator, BRCA2 plays a more significant role in the non-sensitive network, which manifests as two non-sensitive specific (blue) edges in the DDN to NBN and RAD50. In including the two condition-specific nodes, the evidence net acquires a condition-specific bias. We develop this idea further in the discussion below.

## Discussion

In the effort to support the statistical inferences discovered by EDDY, the STITCH/STRING databases provided an abundance of support, which could be filtered using different approaches. Employing a naïve shortest paths strategy, priority was given to the strength of support for edges while minimizing distance between compound and mediator. However, this approach often risked losing the relevance of the original biological pathway used in the EDDY discovery. The coupled effect of a promiscuous compound with a pleiotropic gene could potentially engender numerous unrelated networks. Merging the mediator network with those of its nearest-neighbors aimed to maintain the evidentiary focus of the original EDDY inference.

**Fig. 7:**
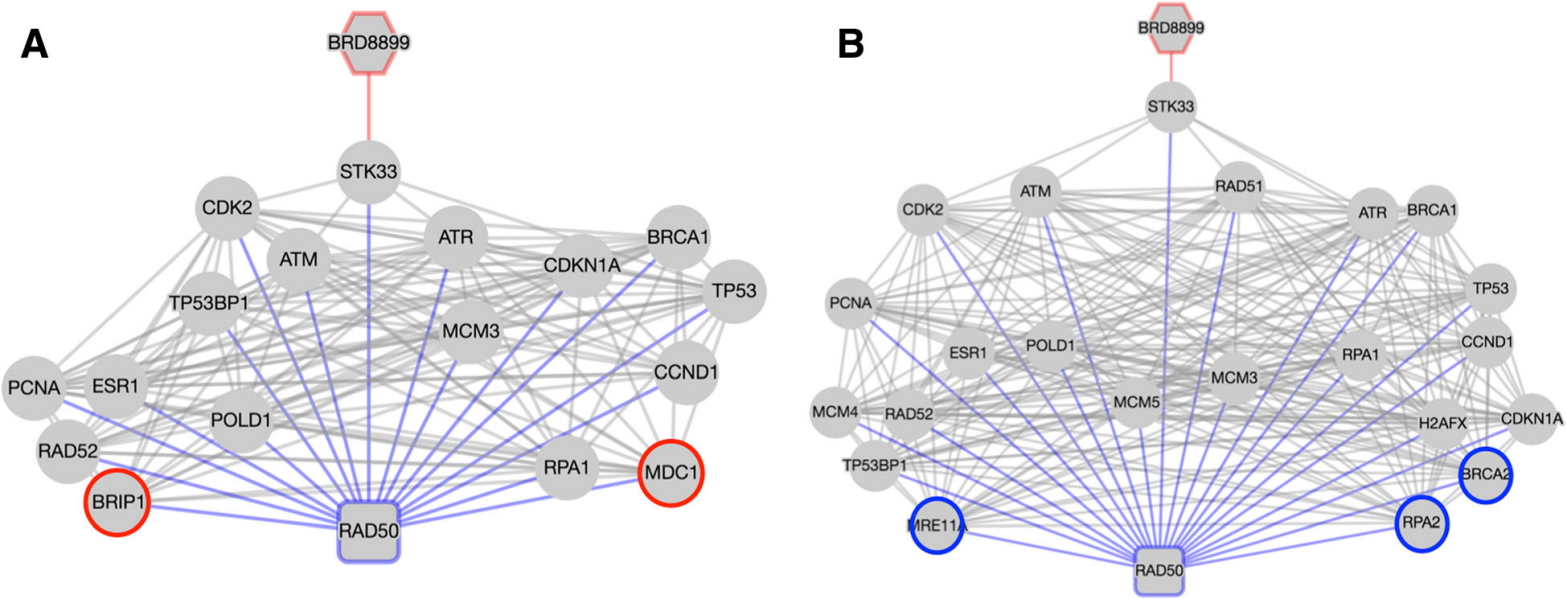
Condition-specific evidence networks for the BRD-8899 – RAD50 compound-mediator pair. Left (**a**) is for the sensitive network and right (**b**) is for non-sensitive. Sensitive (*red*) and non-sensitive (*blue*) specific nodes are indicated by colored border

Despite the increased density of nodes in some of these evidence networks, the inclusion of neighbors allows for the possibility of exploring the nuances of differentiality revealed by EDDY, producing *condition-specific evidence networks*, mirroring the condition-specific dependency networks generated by EDDY. These are particularly compelling in the case of specificity mediators, which have been identified for their significant rewiring between conditions. In Figs. 2 and 5, the specificity mediator RAD50 has edges with BRIP1 and MDC1 for sensitive samples and edges with BRCA2, MRE11A and RPA2 for non-sensitive samples. These neighbors can be integrated into separate condition-specific evidence networks, as shown in Fig. 7.

## Conclusion

In this project, we have improved the interpretability of the EDDY-CTRP results by generating evidence networks of the most relevant intermediate genes using the STRING and STITCH knowledge-bases. With these evidence networks and our Cytoscape.js-based user interface, we expect that the EDDY-CTRP results can be used to form hypotheses based on these contextualized drug-mediator pairs.

Besides facilitating drug-mediator pair interpretation, evidence networks can be used in a more flexible manner, such as when single-ended evidence networks were employed to identify candidate compounds for interaction with a mediator. Furthermore, integration of network information for a mediator’s neighbors can preserve the pathway context of the original DDN. We furthered this synthesis of DDN and evidence network information through the incorporation of condition-specific neighbor nodes.

In the future, we hope to generalize these evidence networks so they can be used with other knowledge bases and with other drug-gene pairs. Other methods such as high-throughput drug screening generate drug-gene hypotheses similar to EDDY-CTRP and would benefit from an algorithmic approach to contextualizing these hypotheses with current research. In future iterations, we aim to use alternative algorithms to Yen’s *K* Shortest Paths such as Eppstein’s *K* Shortest Paths [21] in order to optimize the speed at which the evidence networks are generated. With faster support algorithms, it could be possible to create an interface that would allow researchers to query any drug-gene pair they might be interested in and receive an on-the-fly generated evidence network.
